# CD39/Adenosine Pathway Is Involved in AIDS Progression

**DOI:** 10.1371/journal.ppat.1002110

**Published:** 2011-07-07

**Authors:** Maria Nikolova, Matthieu Carriere, Mohammad-Ali Jenabian, Sophie Limou, Mehwish Younas, Ayrin Kök, Sophie Huë, Nabila Seddiki, Anne Hulin, Olivier Delaneau, Hanneke Schuitemaker, Joshua T. Herbeck, James I. Mullins, Maria Muhtarova, Armand Bensussan, Jean-François Zagury, Jean-Daniel Lelievre, Yves Lévy

**Affiliations:** 1 INSERM, Unite U955, Creteil, France; 2 National Center of Infectious and Parasitic Diseases, Sofia, Bulgaria; 3 Chaire de Bioinformatique, Conservatoire National des Arts et Métiers, Paris, France; 4 AP-HP, Groupe Henri-Mondor Albert-Chenevier, Laboratory of Pharmacology and Toxicology, Creteil, France; 5 Department of Experimental Immunology, Sanquin Research, Landsteiner Laboratory, Center for Infectious Diseases and Immunity Amsterdam (CINIMA) Academic Medical Center, University of Amsterdam, Amsterdam, The Netherlands; 6 University of Washington, School of Medicine, Department of Microbiology, Seattle, Washington, United States of America; 7 INSERM UMR 976 and Universite Denis-Diderot-Paris 7, Hopital Saint-Louis, Paris, France; 8 Université Paris Est Créteil, Faculté de Médecine, Creteil, France; 9 AP-HP, Groupe Henri-Mondor Albert-Chenevier, Immunologie clinique, Creteil, France; Harvard University, United States of America

## Abstract

HIV-1 infection is characterized by a chronic activation of the immune system and suppressed function of T lymphocytes. Regulatory CD4+ CD25^high^ FoxP3+CD127^low^ T cells (Treg) play a key role in both conditions. Here, we show that HIV-1 positive patients have a significant increase of Treg-associated expression of CD39/ENTPD1, an ectoenzyme which in concert with CD73 generates adenosine. We show *in vitro* that the CD39/adenosine axis is involved in Treg suppression in HIV infection. Treg inhibitory effects are relieved by CD39 down modulation and are reproduced by an adenosine-agonist in accordance with a higher expression of the adenosine A2A receptor on patients' T cells. Notably, the expansion of the Treg CD39+ correlates with the level of immune activation and lower CD4+ counts in HIV-1 infected patients. Finally, in a genetic association study performed in three different cohorts, we identified a *CD39* gene polymorphism that was associated with down-modulated *CD39* expression and a slower progression to AIDS.

## Author Summary

HIV-1 infection is characterized by a chronic activation of the immune system. Regulatory T cells (Treg) represent a population of lymphocytes that controls inappropriate or exaggerated immune activation induced by pathogens, thereby influencing the outcome of various infections. Several studies have shown that Treg are expanded in HIV infected patients. However, the mechanisms of Treg immune-modulator functions are not clearly known. CD39 is an ectonucleotidase which converts the proinflammatory ATP signal into AMP and the immunosuppressive adenosine in concert with CD73. A critical role of CD39 has been described for Treg in general but few studies have analyzed its role in HIV infection. We report here an expansion of Treg expressing CD39 in a cohort of HIV-infected patients. In vitro these cells exerted a strong suppressive effect on the effector CD8 T cells. Treg inhibitory effects were relieved by CD39 down-modulation using an anti-CD39 monoclonal antibody. Treg suppressive effects were reproduced by an adenosine agonist in accordance with a higher expression of the adenosine A2A receptor on patients' T cells. From a clinical stand point, we show also a correlation between Treg CD39+ expansion and both immune activation and CD4+ T cell depletion in patients. Finally, by genetic analysis of three different cohorts of patients, we found that a *CD39* gene polymorphism associated with a lower *CD39* expression correlated with a slower progression to AIDS. Thus, our results contribute to elucidate the mechanisms by which Treg suppression occurs during HIV infection.

## Introduction

HIV-1 infection is characterized by chronic immune activation which, in combination with the progressive depletion of CD4+ T cells, profoundly perturbs antigen-specific T cell responses [1]. The population of CD4+CD25^high^ FoxP3+ regulatory T cells (Treg) suppresses antigen-specific T cell responses and controls inappropriate or exaggerated immune activation induced by pathogens, thereby influencing the outcome of various infections [2], [3]. In particular, these cells suppress *in vitro* HIV-1-specific CD4+ and CD8+ effector T-cell responses [2], [4]. We, and others, have reported an HIV-1-driven expansion of Treg expression in chronic and acute HIV-1 infection [5], [6], including a relationship between the expansion of Treg, the level of cellular immune activation and the depletion of CD4+ T cells in acute HIV infection [5].

The molecular mechanisms by which Treg mediate their suppressive activity remain poorly understood. In humans, the Treg population exhibits considerable diversity. Phenotypically and functionally distinct subsets of Treg can mediate suppression through distinct mechanisms from secretion of IL-10, TGF-ß, IL-35, Granzyme B, perforin, to CTLA-4 and GITR interactions [7], [8], [9]. Recently, it has been reported that CD39 is expressed on human and murine Treg, while CD73 is found only on the surface of murine Treg [10], [11], [12]. CD39, a member of the ectonucleotidase triphosphate diphosphohydrolase family (ENTPD), also referred to as ENTPD-1 (EC 3.6.1.5), is the dominant immune system ectonucleotidase that hydrolyses extracellular ATP and adenosine diphosphate (ADP) into adenosine monophosphate (AMP) at the sites of immune activation. CD73 is an ecto-5′-nucleotidase (5′NT) that exists in a soluble or membrane-bound form and catalyzes the dephosphorylation of AMP to adenosine [13], [14], [15]. Adenosine is a critical regulator of innate and adaptive immune responses [16], [17], inhibiting T lymphocyte proliferation and the secretion of inflammatory cytokines including IL-2, TNFa, and IFN-γ [13], [14], [15]. These effects are mediated through A2A receptors stimulating the generation of cAMP, and are mimicked by adenosine agonists [18]. CD39 has also been described as an activation marker of lymphoid cells [19]. Therefore, the CD39/Adenosine pathway may be important to the balance between activation and regulation of effector immune responses.

Here we tested the hypothesis that the CD39/adenosine pathway is involved in the pathogenesis of HIV-1 disease. First, we investigated the phenotype and the function of Treg-expressing CD39 molecules in a cohort of chronically HIV-positive patients and determined whether these characteristics are associated with clinical outcomes. Second, to assess our hypothesis in an *in vivo* context, we investigated whether *CD39* genetic polymorphisms were associated with rates of HIV-1 disease progression in three independent cohorts.

## Results

### CD4+CD25^high^ Treg, but not CD4+CD25^low^ activated T cells, from HIV-positive subjects express high density of cell membrane CD39 molecules

**Figure 1:**
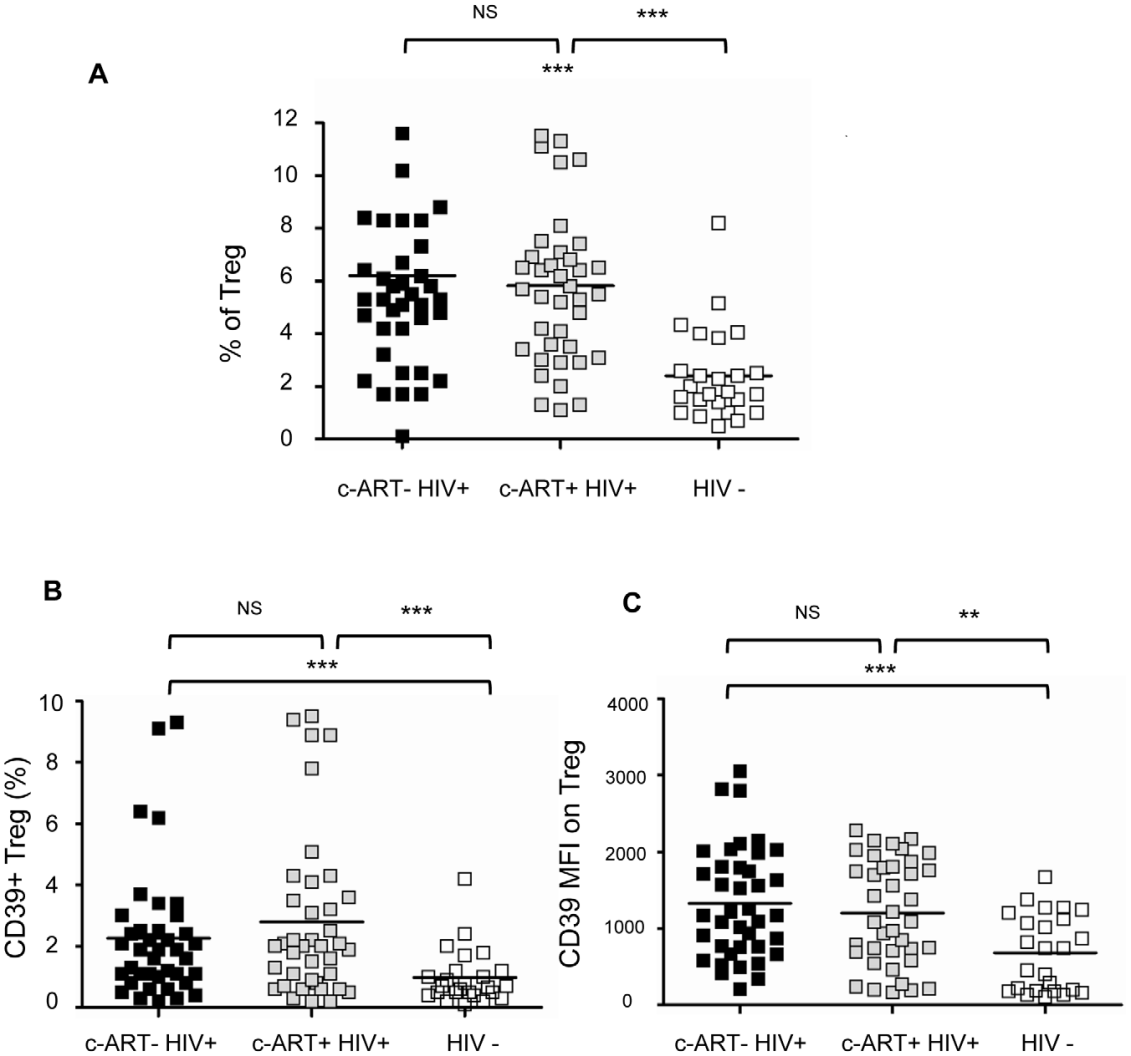
Treg CD39 populations are significantly increased in HIV-1 infected subjects. PBMC from c-ART− HIV-1-positive subjects (black squares, n = 39), c-ART+ HIV-1-positive subjects (grey squares, n = 39) and HIV-negative subjects (white squares, n = 25) were analysed by flow cytometry. The mean (min-max) CD4 T absolute counts were 411 (18–1053) and 650 (117–2523) cells/ml, in c-ART− and c-ART+ patients, respectively. The mean (min-max) plasma HIV RNA values were 4.6 (2.1–6.2) and 1.57 (1.1–2.03) log_10_ HIV-1 RNA copies/ml, in the two groups, respectively. The percentage of CD4^+^CD25^high^Foxp3+CD127^low^ cells (Treg) on CD4 T cells **(A)**, of Treg CD39+ **(B)** and the MFI of CD39 expression on Treg **(C)** are presented. Horizontal lines correspond to the mean for each data set Statistical differences were assessed by unpaired t-test assuming independent samples, ** P<0.01, ***P<0.001.

In order to discriminate between Treg and activated T cells, we further characterized Treg population as gated T cells expressing CD4+CD25^high^ FoxP3+^high^ and CD127^low^ (gating strategy is shown in Fig. S1). These cells are designated thereafter as Treg cells while CD4+CD25^low^CD127^high^ T cells are designated as activated CD4+CD25^low^ T cells (T act). First, we confirmed a significant increase in the percentages of Treg cells in a cohort of HIV-positive individuals, receiving either a combination of antiretroviral drugs (c-ART+, n = 39) or not (c-ART−, n = 39), as compared to healthy controls (n = 25) (mean 5.8% and 6.2% respectively *vs* 2.4%, P<0.0001) (**Fig. 1a**). As shown in **Fig. 1b and 1c**, percentages of Treg expressing CD39+ (Treg CD39+) were significantly higher in both c-ART+ and c-ART− patients, as compared to healthy controls (mean 2.79% and 2.26% vs 0.97%, P<0.001, **Fig. 1b**). Moreover, Treg from both c-ART− and c-ART+ subjects expressed a higher density of CD39 molecules as compared to those from HIV-1 negative controls (mean fluorescence intensity (MFI) 1327 and 1203, respectively, *vs.* 652, P<0.001 and P<0.01) (**Fig. 1c**).

Phenotypic analyses were performed in 16 HIV-1 positive patients before and 12 months following c-ART initiation. Among them, 9 patients experienced a good response to c-ART (group A; undetectable plasma viral load at month 12), while in 7 patients (group B) viral replication remained detectable (above 50 copies/ml). No significant decrease of CD39 expression was observed in group A: % Treg CD39+ (mean ± SD): 2.4±1.2 vs.1.8±1.0 at baseline; TregCD39+ MFI (mean ± SD): 1557±360 vs. 1261±656 at baseline, (P>0.05 for both). Moreover, in patients with on-going viral replication %Treg CD39+ increased significantly in spite of ART (6.1±2.4 versus 3.4±2.3 at baseline; P = 0.043).

CD39 has also been described as an activation marker of lymphoid cells [19]. Therefore, we looked at the percentages of Tact in HIV-1 positive patients and controls. As expected, the frequency of activated CD4+CD25^low^ T cells was significantly higher in both populations of patients as compared to controls (**Fig.S1b**). Consequently, percentages of CD4+CD25^low^CD39+ were significantly higher in HIV-1 positive patients as compared to controls (**Fig.S1c**). In contrast to Treg, CD4+CD25− T cells from both HIV-positive subjects and controls did not express CD39 (not shown). Thus, an expansion of CD39+CD4+ T cells in both Treg and T act T cell populations, which persist in patients with controlled viral load under c-ART, is observed in HIV-1 positive patients. In HIV-positive subjects and in HIV-negative controls, Treg cells were mostly of CD45RA−CD28+ memory phenotype (mean 75%). CD45RA−CD28+ Treg contained a higher percentage of CD39+ cells as compared to CD45RA+CD28+ Treg cells (mean 65% vs. 28%, respectively, P<0.05) (**Fig. S2**).

### Down-modulation of CD39 expression on Treg relieves Treg-mediated inhibition of CD8 T cell proliferation and HIV specific responses

**Figure 2:**
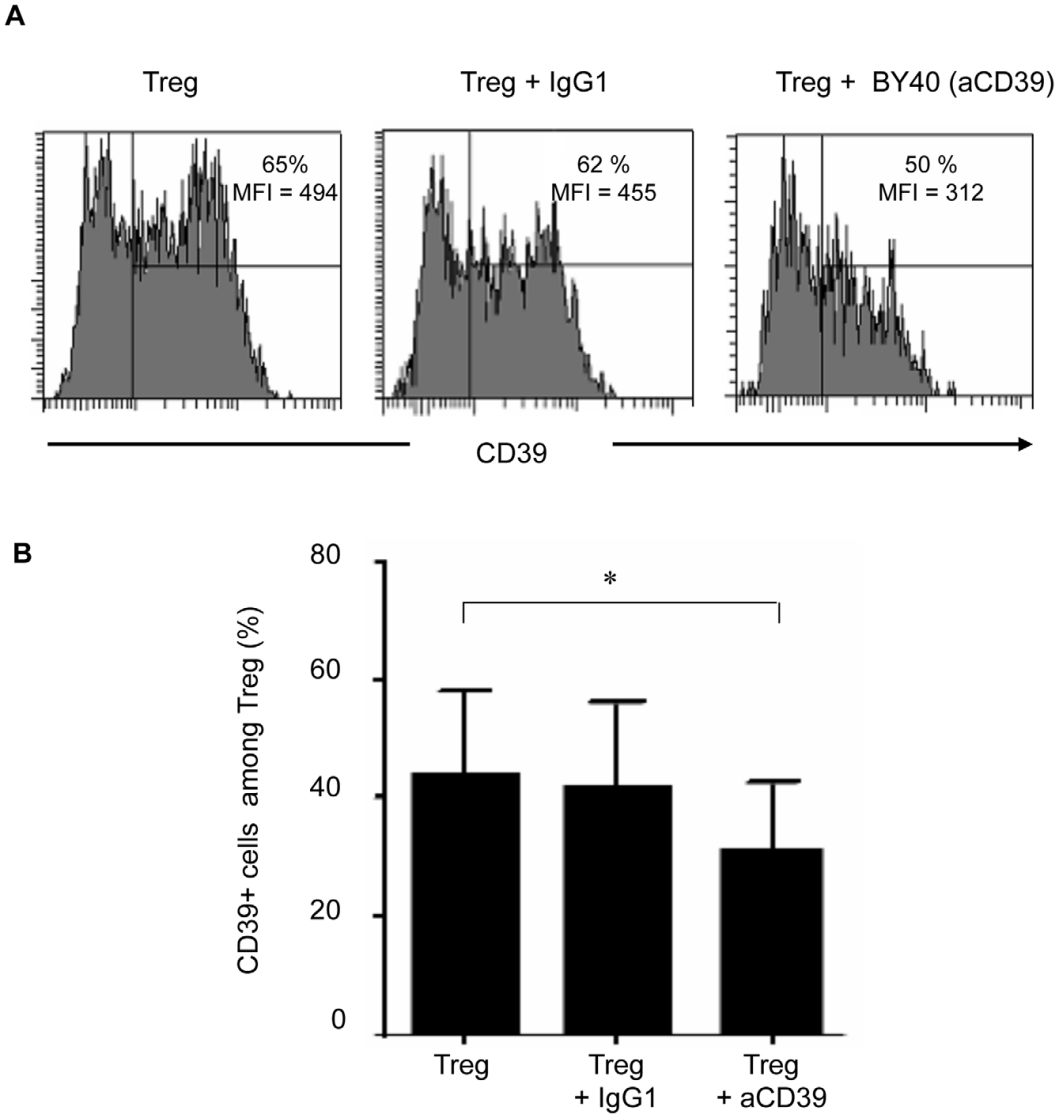
Preincubation of Treg with anti-CD39 BY40 mAb down-modulates CD39 expression on Treg. **(A)** Representative experiment showing the expression of CD39 on Treg from an HIV-negative donor, preincubated in medium alone (left histogram) or either with IgG1 isotype control (middle histogram) or anti-CD39 mAb BY40 (right histogram), and co-cultured for 18 h with anti-CD3-activated autologous CD8 T cells. Expression of CD39 was then assessed using a BY40 non-competitive anti-CD39 mAb on gated CD4^+^CD25^high^CD127^low^Foxp3^+^ cells. **(B)** Pooled data from 3 independent experiments show the percentage of CD39^+^ cells among Treg after co-culture as in **(A)**. Bars represent mean +/− SD. Statistical differences were assessed by a paired Student t-test, * P<0.05.

We next investigated whether down-modulation of the CD39 enzyme can impact Treg function. First, by exposing cells to a blocking anti-CD39 (BY40) mAb, we induced a down-modulation of CD39 expression at the surface of the YT2C2 NK line cells (**Fig. S3a**). Next, BY40 mAb down-modulated the expression of CD39 on *ex-vivo* purified peripheral blood Treg from HIV-negative controls as compared to untreated cells or cells treated with an IgG1 control mAb (% of positive cells (mean ± SD): 32±11% *vs* 44±13%, and 42±14%, respectively) (**Fig. 2a,b**). In these experiments, CD39 expression following in vitro incubation with BY40 mAb was assessed using a commercial PE anti-CD39 (clone TU66) which has been previously checked to be non-competitive with BY40 (**Fig. S4**). Finally, we found that this down modulation effect of BY40 was associated with decreased CD39 ATPase activity on primary monocytes (**Fig. S3b**).

**Figure 3:**
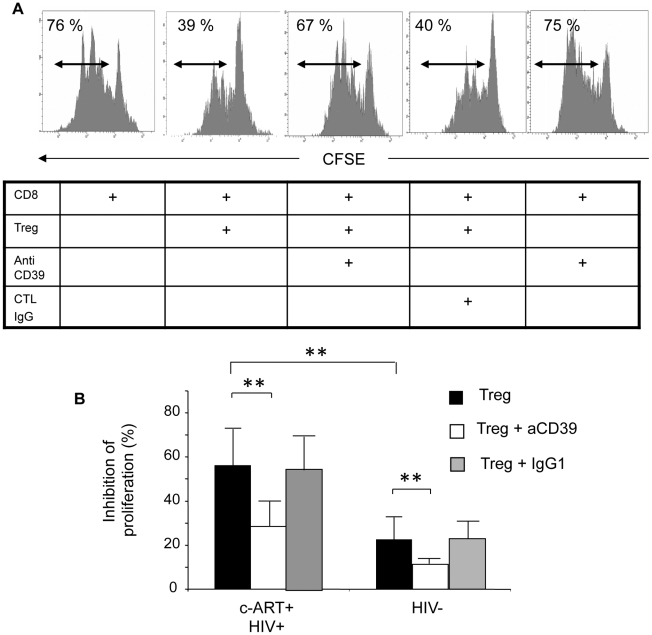
CD39 blocking mAb reverses the suppressive effect of Treg on the proliferation of anti-CD3 stimulated CD8+ T cells. **(A)** Representative histograms showing the anti-CD3 stimulated proliferation of purified CD8+ T alone or co-cultured with Treg without pre-incubation or pre-incubated with anti-CD39 mAb or control IgG1 (Histograms are gated on the CD8^high^ populations). Percentage of proliferating (CFSE^low^) CD8+ T cells is shown for each condition (one representative experiment from 3 performed in triplicate). **(B)** Pooled data showing the percentage of proliferation inhibition of anti-CD3 stimulated CD8+ T cells from c-ART+ HIV-1-positive (n = 6) and HIV-negative subjects (n = 6) in the presence of Treg either alone (black squares), pre-incubated with anti-CD39 mAb (white squares) or with control IgG1 (gray squares). Histograms represent means +/− SD. Statistical differences were assessed by one-way ANOVA followed by a paired T- test, * P<0.05, ** P<0.01.

The functional consequences of CD39 down-modulation were investigated in co-culture assays developed to evaluate the suppressive effects of Treg on T cell proliferation [5], [6], [20]. As shown in **Fig. 3a and b** (for one representative experiment and pooled data from 6 HIV-positive subjects), the Treg-mediated inhibition of anti-CD3 induced CD8 T cell proliferation was significantly higher in HIV-positive subjects (n = 6) as compared to HIV-negative controls (n = 6), (mean inhibition 56% *vs* 22.5%; P<0.01) (**Fig. 3b**). Pre-incubation with anti-CD39 BY40 mAb reversed by ∼50% the suppressive effect of Treg from HIV-positive subjects (average suppression rate of 28% in the presence of Treg pre-treated with BY40 as compared to 56% and 57% for Treg pre-treated or not with IgG1 control mAb, (P = 0.01; one-way ANOVA and paired T-test P = 0.01 for group by group comparisons). Interestingly, although the suppression mediated by Treg from HIV-negative controls was less significant, a similar effect of anti-CD39 BY40 mAb was noted (average inhibition 12.3% as compared to 22.5%, one-way ANOVA P<0.01 and paired T-test P<0.01). These results are in accordance with the higher density of CD39 molecules expressed by Treg from HIV-positive subjects and indicate that this enzyme is involved, at least in part, in the Treg-mediated inhibition of CD8+ T cell proliferation.

**Figure 4:**
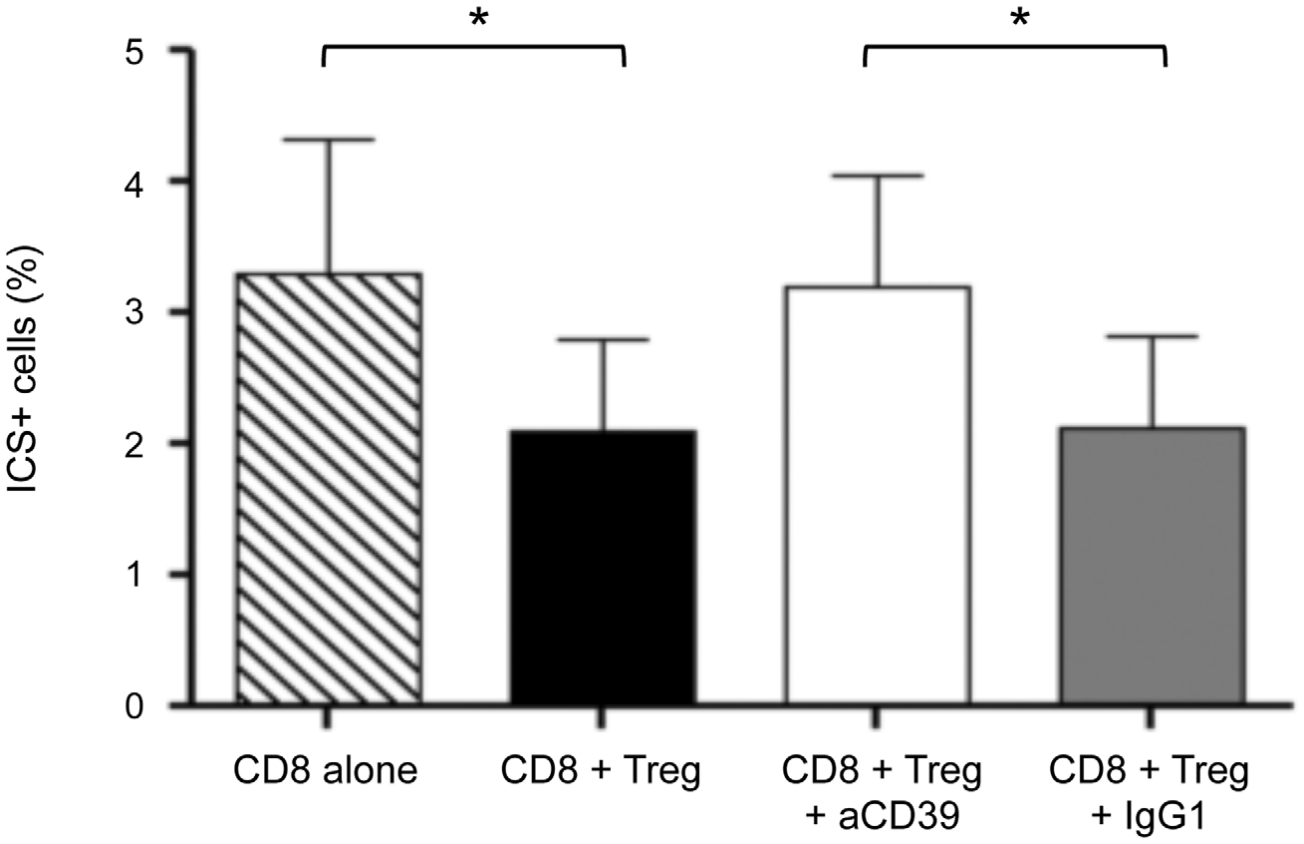
CD39 blocking mAb reverses the suppressive effect of Treg on the cytokine production of Gag-stimulated CD8+ T cells. Pooled data showing the percentage of CD8+ T cells from c-ART− HIV-1-positive patients (n = 5) producing cytokines (IL2/IFN-g/TNF-a) after overnight stimulation with Gag peptides. CD8+ T cells were cultured in the presence or not of Treg (ratio ¼) and in the presence or not of antiCD39 Mab or isotype control (see M&M). Histograms represent means +/− SD. Statistical differences were assessed by a paired t-test, * P<0.05.

Next, we evaluated the effects of Treg on the cytokine production of CD8 T cells in response to HIV-1 antigens. Cytokine production (IFN-γ, TNFα and IL-2) of CD8-gated T cells was analyzed by intra cytoplamic staining and flow cytometry after overnight stimulation with a pool of whole Gag 15mer peptides (2 µg/ml). As shown in Fig. 4, the percentages (mean ± SD) of CD8+ Cytokines+ T cells were 2.1+/−0.7% vs. 3.3%+/−1% (n = 5) in the presence of Treg and CD4+CD25− respectively (P = 0.05). Pre-incubation of Treg with anti-CD39 mAbs, but not with isotype control, relieved this suppressive effect: 3.2+/−0.8%, (P = 0.05).

Together, these results indicate that CD39 enzyme participates in the Treg-mediated suppression on CD8 T cell proliferation and responses to HIV peptides.

### T cells from untreated HIV-positive subjects are more susceptible to CD39/adenosine mediated inhibition due to increased expression of A2A receptor

**Figure 5:**
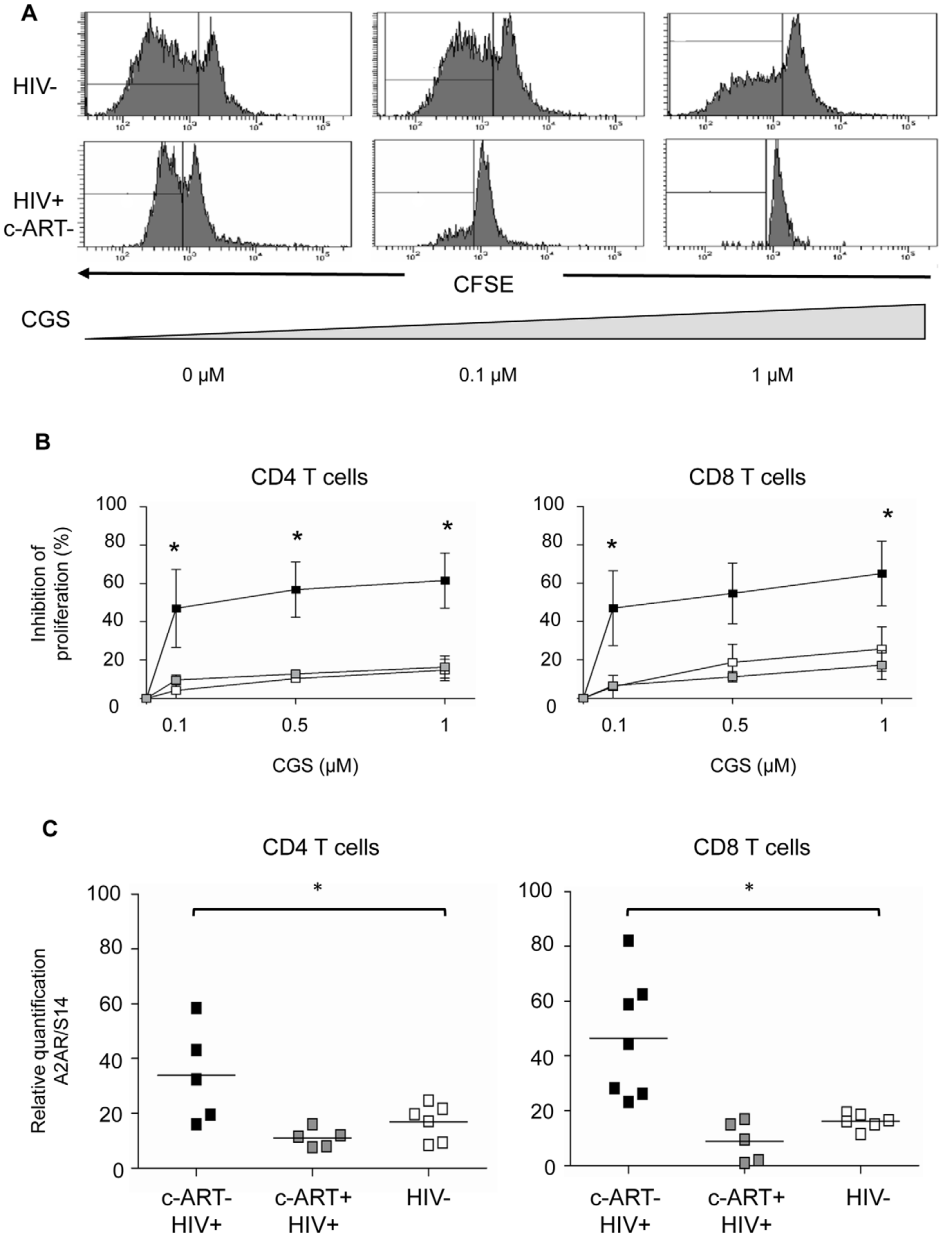
T cells from c-ART− HIV-1 positive patients are more susceptible to the inhibitory effects of the adenosine agonist CGS-21680 and express a high density of A2A receptor. **(A)** PBMC from one representative control (upper panel) and one c-ART− HIV-1-positive subject (lower panel) were labelled with CFSE and activated using anti-CD3 mAb. CGS-21680 was added at day 0 and histograms of CFSE staining of gated CD8+ T cells are from day 5. **(B)** Pooled data (n = 4) from c-ART− (black squares) and c-ART+ (grey squares) HIV-positive, and from HIV-negative (white squares) subjects showing the dose-dependent effect of CGS-21680 on the proliferation of anti-CD3 activated CD4+ (left panel) and CD8+ (right panel) T cells treated as in **(A)**. **(C)** CD4+ and CD8+ T cells were purified from the blood of c-ART− subjects (black squares, n = 5), c-ART+ subjects (grey squares, n = 7), and HIV-negative subjects (white squares, n = 6). A2AR mRNA expression was assessed using qPCR. Results were standardized using the expression of the S14 mRNA house-keeping gene. Horizontal lines correspond to the mean for each data set, statistical differences were assessed by one-way ANOVA and unpaired t-test assuming independent samples, * P<0.05., * P<0.05.

To further investigate the involvement of CD39/adenosine in the Treg-mediated inhibition of CD8+ T cell proliferation in HIV-1 positive subjects, we studied the effects of the A2AR agonist CGS21680 on proliferation of anti-CD3 stimulated T cells. The mean (±SD) inhibition of CD4+ T cells was 47% (±11) and 57% (±8.3) in the presence of 0.1 and 1 mM of CGS, respectively in c-ART− HIV positive patients. Similarly, the same doses of CGS inhibited by 47% and 65% the proliferation of anti-CD3 activated CD8+ T cells from c-ART− HIV-positive subjects (P<0.05) (**Fig. 5a,b**). In contrast, the proliferation of CD4+ and CD8+ T cells from HIV-negative controls and c-ART+ HIV-positive subjects was much lower and below 20% at the highest dose of CGS21680 (1 mM) (**Fig. 5a,b**) (P = 0.015 and P = 0.027 respectively; one-way ANOVA and P<0.05 unpaired T-test for comparison between c-ART−HIV-positive patients and the two other groups (**Fig. 5a,b**).

In accordance with this, we found that both CD4+ and CD8+ purified T cells from c-ART− HIV-positive subjects (n = 7) expressed a significantly higher level of A2AR mRNA than c-ART+ subjects (n = 5) or HIV-negative controls (n = 6) (**Fig. 5c**).

### Expansion of Treg CD39+ correlates directly with immune activation and inversely with CD4+ T cell absolute counts in HIV-positive subjects

**Figure 6:**
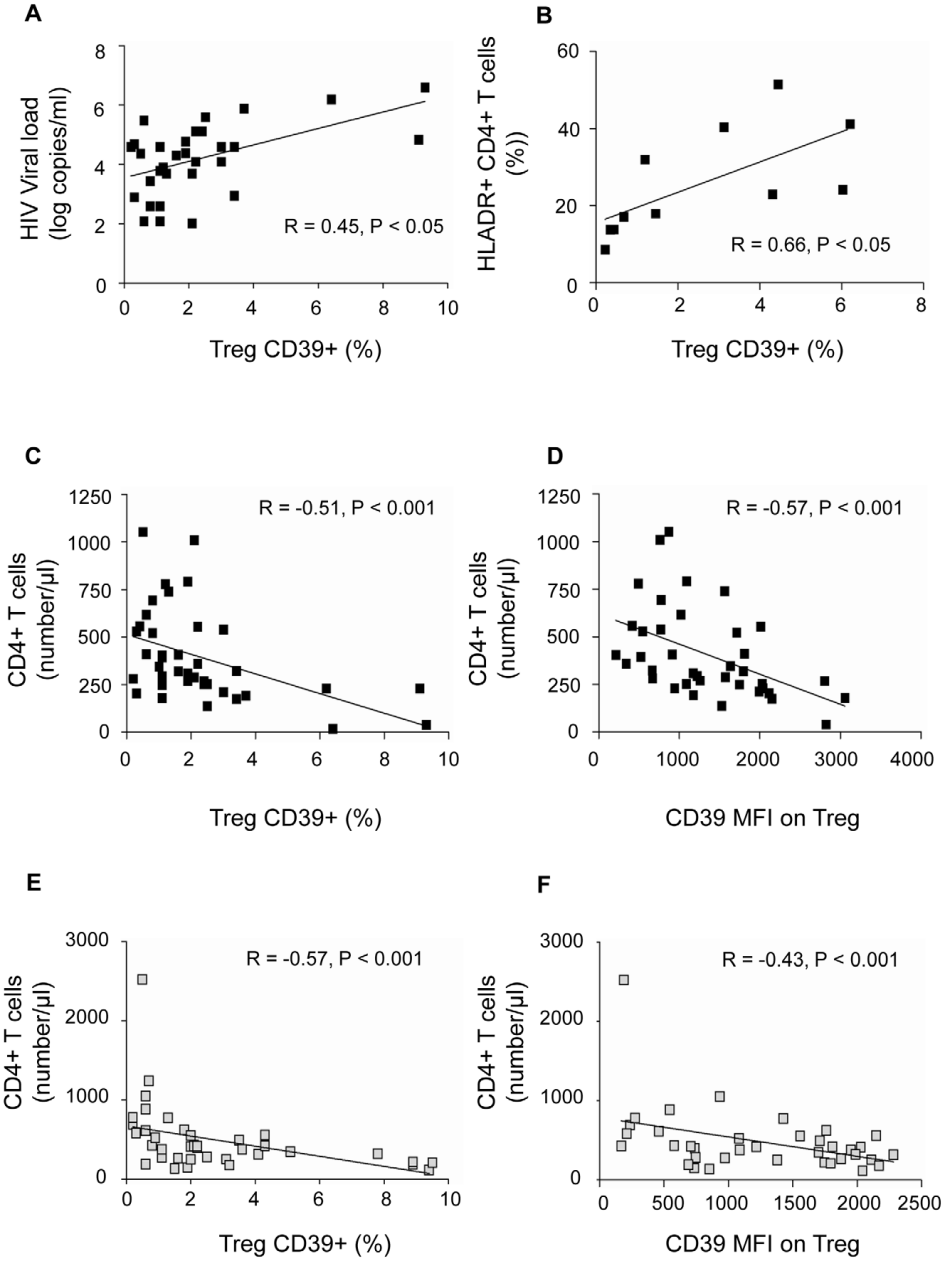
CD39 expression on Treg correlates positively with viral load and T cell activation and negatively with CD4+ T cell count in HIV-1 positive subjects. Data from c-ART− (A, n = 31, B, n = 11, C, n = 39, D, n = 38 and, black squares) and c-ART+ HIV-1 subjects (E, n = 39, F, n = 37, gray squares) are shown. In c-ART−HIV-1 positive subjects, the percentage of Treg CD39+ correlates directly with HIV-1 viral load (A) and the expression of the activation marker HLA-DR on CD4+ T cells **(B)**. The percentage of Treg CD39+ **(C, E)** and the MFI of CD39 expression on Treg **(D, F)** correlates inversely with the absolute CD4+ T cell count in both c-ART+ and c-ART− groups. Correlations were assessed using Spearman's rank order test.

Since the HIV-positive subjects we studied were heterogeneous in terms of disease duration and clinical stage, we assessed whether CD39 expression correlated with established markers of disease progression. The frequency of the Treg CD39+ subset correlated directly with plasma HIV-1 viral load in the group of c-ART− subjects (P<0.05, R = 0.45) (Fig. 6a). Moreover, the percentage of Treg CD39+ subset correlated directly with the activation of CD4+ T cells in c-ART− subjects, assessed by the percentage of CD4+HLA-DR+ (P<0.05, R = 0.66) (Fig. 6b). Finally, the percentage of Treg CD39+ cells and CD39 MFI correlated inversely with absolute CD4+ T cell count in c-ART− subjects (P<0.001, R = −0.51 and P<0.001, R = −0.57, respectively) (Fig. 6 c,d) as well as in c-ART+ subjects (P<0.001 , R = −0.57 and P<0.01, R = −0.43) (Fig. 6 e,f).

The independent prognostication value of CD39 expression on Treg for CD4 T cell counts was studied in c-ART− and in c-ART+ patients, using multiple linear regression models (SPSS v.17.0). The frequencies of Treg, Treg CD39+, Tact, Tact CD39+, and viral load (for c-ART− group only) were included as predictors of CD4 absolute count. For c-ART+HIV-positive patients, in a full model (R^2^ = 0,398, ANOVA P = 0.02) the percentage of Treg CD39+ had the most important partial predictive effect (partial correlation coefficient −0.479), confirmed by sequential multiple regression analysis of the same set of variables (partial correlation coefficient −0.612 vs. 0.360 for Tact, ANOVA P = 0.001).

For c-ART−HIV-positive patients, in a full model (R^2^ = 0,392, ANOVA sig. = 0.045), again the percentage of TregCD39+ had the most important contribution as predictor for CD4 absolute count, followed by CD39Tact (partial correlation coefficients −0.375, and 0. 265 respectively).

These results indicate that the frequency of Treg CD39+ is an independent predictive factor for CD4 cell count variability.

### A genetic variant of *CD39* associated with a lower gene expression is involved in slower progression to AIDS

Our results highly suggest that the frequency of Treg CD39+ cells, as well as the density of the enzyme molecule at the surface of those cells, predict disease progression. Recently, *CD39* gene polymorphisms associated with the level of enzyme expression have been shown to be associated with susceptibility to Crohn's disease [21]. In order to assess the role of CD39 on HIV-1 disease progression, we investigated whether *CD39* gene polymorphisms could be associated with clinical outcomes. For that, we exploited the GRIV cohort, comprising subjects exhibiting extreme profiles of AIDS progression (LTNP, long-term non-progressors and RP, rapid progressors) [20], [22], [23]. We thus performed a genetic case-control association study on the candidate gene *CD39* using the genotype data collected from our previous genome-wide association studies [22], [23] (see Methods).

Fourteen SNPs were identified in the *CD39* gene. No polymorphism was significantly associated with rapid progression, whereas four SNPs were significantly associated with LTNP: rs10882665 (P = 1.33×10^−2^), rs3181123 (P = 1.38×10^−2^), rs1933166 (P = 1.76×10^−2^), and rs11188513 (P = 3.60×10^−2^) (**Fig. S5**). Of note, rs10882665 and rs3181123 are in full linkage disequilibrium (*r^2^* = 1). To eliminate a potential association with HIV-1 infection rather than with LTNP, we compared the allelic frequency of each of these SNPs in the RP population. The frequency observed in the RP group was similar to the frequency observed in the control group, confirming that this was an association with LTNP.

**Figure 7:**
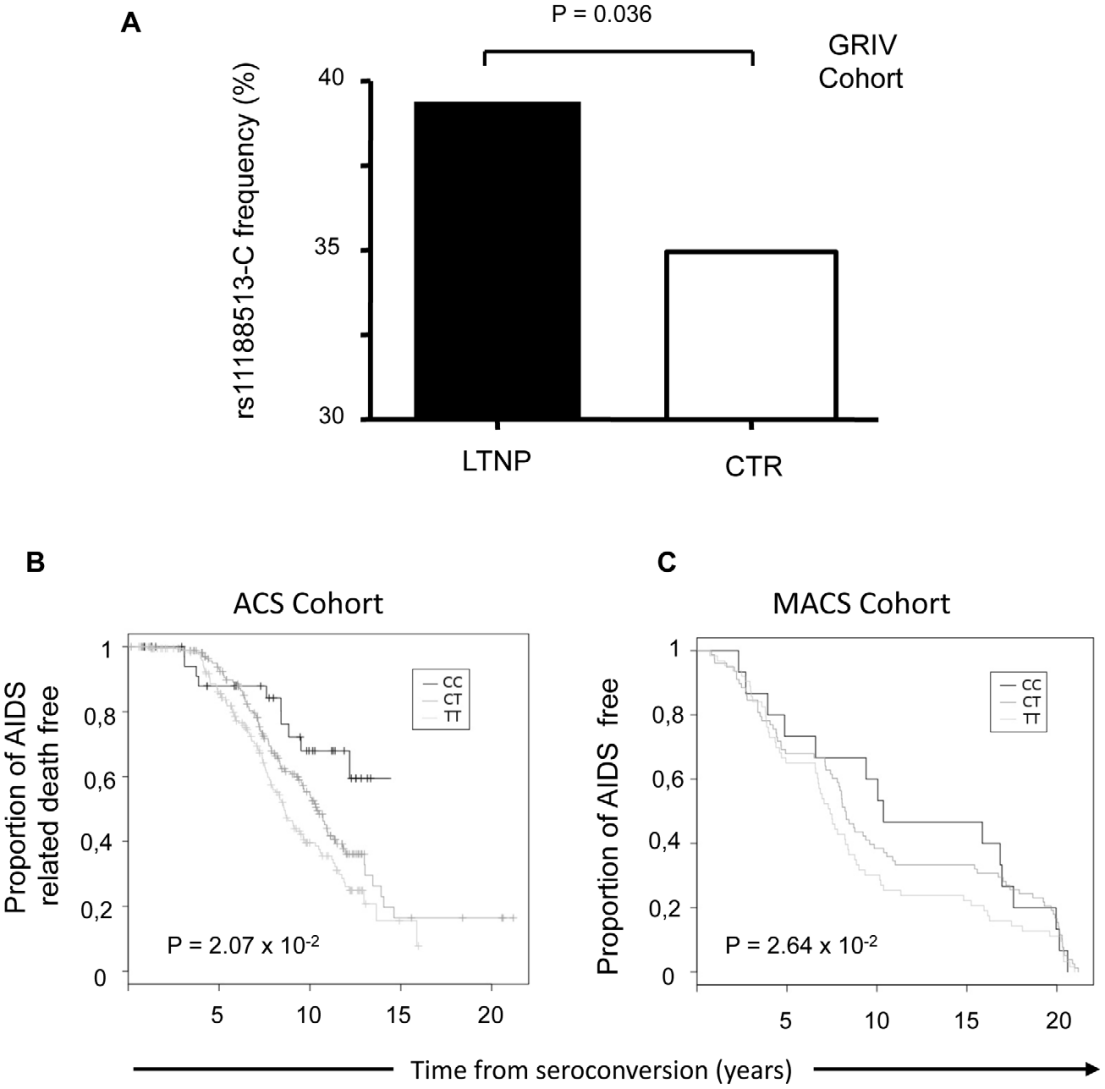
Effect of rs11188513 in the GRIV, ACS and MACS study groups. **(A)** Allelic frequency of rs11188513-C in the GRIV LTNP population (LTNP, n = 275) and the control group (CTR, n = 697). **(B)** Kaplan-Meier survival curve derived from the ACS cohort (n = 404) for time to AIDS-related death. Genotypes CC (black), CT (dark grey) and TT (light grey). **(C)** Kaplan-Meier survival curve derived from the MACS cohort (n = 156) for time to clinical AIDS. Genotypes CC (black), CT (dark grey) and TT (light grey). P-values were computed by regression in an additive model including as covariates the 10 principal Eigenstrat components. The GRIV cohort comprised 275 LTNP and 86 RP French HIV-1 seropositive individuals of Caucasian descent. The control group comprised 697 French HIV-1 seronegative individuals of Caucasian descent from the D.E.S.I.R. program. In the ACS cohort, 417 HIV-1 subjects were collected on the course of HIV-1 infection using AIDS-related death as an endpoint. In the MACS cohort, 156 HIV-1 Caucasian homosexual men were included, using time to clinical AIDS as an endpoint.

To confirm these results, we used two additional independent Caucasian cohorts that examined AIDS progression phenotype: the ACS and the MACS cohorts (see Methods). The rs11188513 SNP (whose frequency in LTNP and control groups were, respectively, 39% and 34%, P = 3.60×10^−2^, (**Fig. 7a**) was the only polymorphism also associated with disease progression both in ACS (P = 2.64×10^−2^) and MACS (P = 2.07×10^−2^) (**Fig. 7b,c** **and Table S1**). The P values compute the probability that an association is due to chance and the combined P value for rs11188513 over the three cohorts was significant after Bonferroni corrections, *P* = 6.11×10^−3^. Importantly, as shown in **Fig. 7**, the rs11188513-C allele favoured slower progression of HIV infection in all three cohorts. This association was independent from the CCR5 polymorphisms (P1 and Delta32) also located in chromosome 3, since the p value was not modified by using the CCR5 variants as covariates.

To further explore this association, we examined the *Genevar* [24] and the Dixon [25] mRNA expression databases, and found a correlation (P = 3.26×10^−5^ and P = 1.9×10^−14^, respectively) between the rs11188513-C allele and lower expression of the *CD39* gene. Thus, the genetic association study combined with the mRNA expression database information demonstrate that the rs11188513-C allele is associated both with a slower progression to AIDS and with a lower expression of *CD39* gene.

## Discussion

We show here the involvement of the CD39/adenosine pathway in the Treg-mediated suppressive effect on HIV-1-infected subjects' T cell functions. We demonstrate that HIV-positive subjects exhibit both a higher frequency of Treg CD39+ and a higher *in vitro* sensitivity of effector T cells to the suppressive effect of adenosine, due to a higher expression of its predominant A2A receptor. Expansion of Treg CD39+ correlates inversely with CD4 T cell counts in HIV infection independently of plasma viral loads and T cell activation. Finally, in a genetic association study conducted in three different HIV-positive cohorts we show that the level of *CD39* gene expression can indeed impact the course of disease progression.

Recent data have shown that mouse Treg constitutively express CD39 [26], while the proportion of Treg CD39+ cells appears highly variable in healthy human controls [10]. Therefore, in contrast to mice, CD39 expression might delineate a subpopulation of human Treg [10], [27]. However, studies on human Treg CD39+ cells are scarce. Few studies have analyzed the expression of CD39 in HIV disease [28]. Leal et al. have shown an increased nucleotidase activity related to enhanced CD39 expression on lymphocytes of HIV-positive subjects [28]. More recently, and in accordance with results presented here, an increase in the frequency of Treg expressing CD39 has been shown in different cohorts of HIV infected patients [29]. However, these observations warrant further investigations on the role of CD39 and the clinical relevance of these findings. Our results reinforce these observations and provide new insights about the biological mechanisms involving the CD39/adenosine axis. The demonstration that blocking of CD39 with BY40 mAb relieved, although not completely, the suppressive effect of Treg on effector T cells opens the way to new therapeutic interventions aimed to modulate Treg functions [29]. Moreover, we found that Treg CD39+ inhibit cytokine production by HIV-specific CD8 T cells, an effect partially relieved by pre-incubation of Treg CD39+ with anti-CD39 mAb. These results demonstrate that CD39 enzymatic pathway is responsible, at least in part, for the inefficiency of CD8 T cells responses in chronic HIV-1 infection. In contrast, the CD39 pathway seemed to be less predominant in coculture studies performed with cells purified from HIV negative controls. However, we cannot rule out that down-modulation of CD39 enzymatic activity may also interfere with other suppressive pathways.

Our results are similar to those reported in cancer and HIV patients in whom the purified Treg CD39+ subset mediated a higher suppression as compared to control patients [27]. From a clinical stand-point, it is interesting to note the persistence of a higher frequency of Treg CD39+ cells in HIV-positive subjects with controlled viral load, as compared to HIV-negative controls. Likely, this may reflect ongoing chronic immune activation. We show here that the frequency of TregCD39+ is correlated positively to the percentages of activated CD4+ T cells expressing HLA-DR (Fig. 6b) and a higher frequency of conventional T cells (CD4+CD25−) expressing CCR5 (not shown) which may partly explain CD4+ T cell depletion. Alternatively, since the Treg CD39+ subset is mostly confined to the memory CD4 T cell compartment, this population may represent HIV-inducible Treg, as previously reported [5], [6]. Recently, an expansion of suppressive FoxP3+CD39+ CD8 regulatory T cells associated with poor antiviral response has been reported in HIV-infected patients [30]. In our study, we have checked that expression of CD39 molecule on other blood subsets (B, NK and monocytes) did not vary significantly between patients' groups (Fig S6).

Altogether these results support the conclusion that the Treg subset expressing a high density of both CD25 and CD39 molecules represents a highly-enriched population of suppressor T cells in HIV-1 infected patients.

Adenosine is formed in tissue microenvironments under inflammatory insult [16], [31], [32], [33]. Several studies have shown that adenosine plays an important non-redundant role in the regulation of T cell activation [18], [34], [35]. Using the dose-dependant inhibitory effect of the adenosine receptor agonist CGS21680 [18], we confirmed the involvement of CD39/adenosine pathway in the Treg-mediated inhibition of T cell proliferation in HIV-1 infected patients. It is noteworthy that CD39/adenosine inhibition affected both CD8 and CD4 T cells, and was significantly more important in c-ART-naïve HIV positive subjects. This latter difference was due to a significantly higher level of A2AR expression. We found that CGS21680 did not inhibit the proliferation of T cells from c-ART treated patients. However, as we did not evaluate CGS21680 effects on other T cell functions, we cannot rule out that A2AR agonists may also impair T cell cytotoxicity and production of cytokines such as IL-2 and IFN-g rather than cell proliferation, as recently demonstrated [36], [37].

Our data provide clues to the suppressive mechanisms of Treg in the context of chronic immune activation. CD39 expression by Treg is important for the extracellular removal of ATP and allows Treg infiltration of inflamed tissues, resulting in an increase of local extracellular adenosine concentration by ATP catabolism [11], [38]. Extracellular ATP depletion may also increase Treg survival and favour the local accumulation of Treg, since high levels of ATP have been shown to be a pro-apoptotic factor [39], [40]. On the other hand, this microenvironment represents a self-protective mechanism against immune attacks [16], [41] by inducing a rapid tolerization of activated cells, as demonstrated in cancer models [42]. Recent data in a mice model has shown that tissue-derived adenosine promotes peripheral tolerance by inducing T cell anergy and Treg differentiation [37]. Altogether, these studies show that initiation of T cell activation in inflamed tissue and/or tumour microenvironments might result in the induction of T cell unresponsiveness by an A2AR-dependent mechanism. These observations may explain the reports of HIV infection in which Treg coexist in tissues infiltrated with HIV-specific T cells that are poorly capable of controlling local HIV replication [43], [44]. Of note, our study was limited to peripheral blood. Whether, the involvement of CD39/adenosine pathway plays also a key role in secondary lymphoid organs or in mucosa deserves further studies.

Treg CD39+ expansion may help establish the relationship between immune activation and Treg-mediated suppression in HIV-1 infection. Increased ATP and adenine nucleotides in inflamed sites may serve as substrates for Treg-expressed nucleotidases but also may exert direct Treg-activating effects [45]. Thus, the ATP-Treg balance might be crucial for the regulation of inflammation. However, in the long term, CD39-mediated inhibition of T cell proliferation might exert an adverse effect not only on the immediate generation of T-cell immune responses, but also on the maintenance and restoration of the T-cell pool, thus contributing to disease progression. We also showed that despite efficient c-ART, the percentage of Treg CD39+ remains higher in c-ART+ HIV-1 subjects as compared to controls. Although T cells from these individuals express low levels of A2AR, we found that Treg still exert a significant inhibitory effect that was relieved by anti-CD39 blocking antibodies. This observation corroborates the observation of an inverse relationship between the frequency of Treg CD39+ and CD4+ T cell counts in patients (**Fig. 6**). Although the role of Treg in HIV-1 infection remains unclear, the identification of a novel Treg subset participating in Treg suppression may be useful to discriminate between a “friend or foe” role of Treg in HIV-1 infection.

Through a candidate gene association study, we identified a *CD39* gene variant associated with down-modulation of *CD39* expression that impacts the course of disease progression, a finding that was replicated in three different cohorts. Such high P values for the association of this variant and *CD39* expression in both *Genevar* and Dixon databases are extremely rare. Since the SNP identified is in high linkage disequilibrium (*r^2^*>0.9) with several other SNPs within the *CD39* gene, further studies are warranted to determine which of them is a causal variant. It is important to note that, according to the HapMap database, this SNP exists at a allelic frequency of ∼30% in the African population and at ∼70% in the Asian population, suggesting that this genetic variant may be an important determinant of disease progression in both populations. Overall, the genetic association study confirms *in vivo* the hypotheses put forward by our experimental work: subjects carrying the *CD39*-C allele are likely to exhibit a lower *CD39* expression, which could impact the control of T cell immune responses, and in turn slow down HIV-1 disease progression.

Our data show that the CD39/adenosine axis might be a novel pathway involved in the Treg-mediated suppression in HIV infection through both an expansion of Treg strongly expressing the ectonucleotidase CD39, and an increased sensitivity of patients' T cells to adenosine. In this context, the possibility to revert Treg-mediated inhibition using CD39-blocking mAb or by modifying the adenosine turnover with specific drugs seems an attractive approach for the design of novel treatments to enhance T lymphocyte restoration and effector T cell responses.

## Materials and Methods

### Patients and cell populations

Blood samples were collected from HIV-1-positive subjects either naive from treatment (c-ART–, n = 39, CD4+ T cells counts (mean ± SD): 387±242 cells/µl; viral load (mean ± SD): 4,2±1,1 log HIV RNA copies /ml or stable under c-ART for more than 6 months (c-ART+, n = 39, CD4+ T cells counts (mean ± SD): 485±440 cells/µl ; viral load <1,6 log copies /ml), at the Hospital of Infectious Diseases, Sofia, Bulgaria and Henri Mondor Hospital, Créteil, France. Blood from 25 HIV-negative donors was obtained at the Regional Blood Transfusion Centre, Creteil, France. CD8+ and CD4+ T cells were purified using RosetteSep enrichment antibody cocktails (StemCell Technologies, Vancouver, BC, Canada) according to the manufacturer's instructions. CD4+CD25^hi^ cells were further isolated with CD25 magnetic beads and two passages on MS columns (Miltenyi Biotec, Bergisch-Gladbach, Germany). The positive fraction contained >80% Treg expressing high levels of FoxP3 transcription factor as verified by flow cytometry (data not shown).

### Proliferation and intra-cellular cytokine production assays

CD8+ T cells were stained with 0.5 mM CFSE (Molecular probes, Eugene OR, US) as previously described [46]. CFSE-labelled CD8+ T cells were cultivated in 96-well U-bottom plates, coated with 5 mg/mL anti-CD3 mAb (UCHT1; Beckman Coulter, Villepinte, France) in the presence or absence of Treg (total cell concentration 1.25×10^5^/ml and final volume 200 ml and the Treg/Effector ratio was 1/4 as determined in previous studies [43], [44]). In some experiments, Treg were pre-incubated with 10 µg/ml of anti-CD39 (BY40, IgG_1_) or isotype control mAb for 15 min at 37°C, and added to CD8+ T cells without a washing step.

The effects of BY40 mAb on CD39 expression and inhibition of ATPase activity were evaluated using YT2C2 NK cell line (flow cytometry) and fresh monocytes using malachite green phosphate detection kit (R&D System, Minneapolis, USA), according to manufacturer's instruction (See methods in the legend of Fig. S3). To assess the effect of adenosine analogue CGS 21680, PBMC were pre-incubated for 1 h with different concentrations of either CGS 21680 (Sigma-Aldrich, Lyon, France) or DMSO as control. Cells were then stimulated with anti-CD3 for 5 days as described above. At day 2 of culture, DMSO and CGS 21680 were added in identical concentrations.

For intracellular staining (ICS), CD8+ T cells were stimulated in the presence or absence of Treg (Treg/effector ratio:1/4) overnight with a pool of whole Gag 15-mer peptides (2 µg/ml) supplemented with anti-CD28 and anti-CD49d antibodies (1 µg/ml of each). Brefeldine A (10 µg/ml) was added 1 h after the peptide stimulation. Cells were surface stained with anti-CD8 mAb and ICS was performed with PE-Cy7-conjugated IFN-γ, TNFα and IL-2 antibodies. When indicated, Treg were pre-incubated with 10 µg/ml of anti-CD39 mAb or isotype control for 15 min at 37°C, and added to CD8+ T cells without a washing step.

### A2AR mRNA quantification

Total RNA was isolated from purified CD4+ and CD8+ T cells and RT-PCR was performed by the ABI Prism 7500 Sequence Detection System (Applied Biosystems, Courtaboeuf, France) in 50 µL reaction with Platinum SYBR Green qPCR SuperMix-UDG w/ROX (Invitrogen) and 0.2 µM of each primer. S14 mRNA which expression was found to be stable among the different group of patients was used as control to normalize each sample. Sequences of the A2AR- and S14-specific primers were forward: CGAGGGCTAAGGGCATCATTG, reverse: CTCCTTTGGCTGACCGCAGTT) and forward: GGCAGACCGAGATGAATCCTCA, reverse: CAGGTCCAGGGGTCTTGG TCC. The relative levels of A2AR mRNA were calculated using the 2^−ΔΔ*C*T^ method.

### Immunofluorescence and flow cytometry

Anti-CD39-PE (clone TU66), anti-CD25-PC7, anti-CD4-FITC or Pac.blue, anti-CD8-PerCP, anti-CD3-APC, and CD28-PerCP-Cy5.5, were products of BD Biosciences (Le Pont de Claix, France),CD45RA-ECD from Beckman Coulter (Villepinte, France), and CD127-Biot/ strepta-APCCy5.5, FoxP3-Alexa 488, CCR7-APC-Alexa 750 from ebiosciences (Montrouge, France). Blocking anti-CD39 mAb (BY40) was produced in one of our laboratories (A.B) by immunizing mice with the YT2C2 NK cell line. BY40 is IgG1 monoclonal antibody, which is with BY12 mAb unique regarding its epitope mapping as we previously reported [47]. BY40 is not cytotoxic and it inhibits directly ATPase activities mediated by cell membrane anchored CD39 (AB personal data and this paper Fig. S3) Cells were analysed by LSR II (BD Immunocytometry systems). At least 20 000 CD4 or CD8-gated events were collected for cell surface studies.

### Statistical analysis

Statistically significant differences were assessed by one-way ANOVA, followed by paired t-samples T-test, or by unpaired T-test assuming independent samples where appropriate. Correlations were assessed using Spearman's rank order test (GraphPad° Prism 5.0 statistical software). The independent prognostication value of CD39 expression on Treg was evaluated in multiple linear regression models (SPSS v.17.0).

### Cohorts used for the CD39 genetic association study

#### The GRIV cohort

The GRIV (Genomics of Resistance to Immunodeficiency Virus) cohort comprised 275 LTNP and 86 RP French HIV-1 seroprevalent individuals of Caucasian descent [20], [48]. All of them are French HIV-1 seroprevalent subjects of Caucasian descent and included on the basis of the main clinical outcomes, CD4 T-cell count and time to disease progression: an asymptomatic HIV-1 infection for more than 8 years, no antiretroviral treatment and a CD4 T-cell count consistently above 500/mm^3^ for LTNP; and a drop of CD4 T-cell count below 300/mm^3^ less than 3 years after the last seronegative test for RP. The control group used for comparison with GRIV subjects [22], [23] comprised 697 French HIV-1 seronegative individuals of Caucasian descent from the D.E.S.I.R. program [49]. GRIV is a case-control study (LTNP *vs.* CTR or RP *vs.* CTR) and the comparison of the LTNP *vs.* RP groups is less adequate to identify signals for the following reasons: 1. The past experience has shown that most signals are either linked to long-term non-progression or to rapid progression, very rarely to both; 2. Without a control population, the LTNP *vs.* RP comparison does not allow to discriminate if a putative signal is associated to LTNP or to RP; 3. Finally, the three groups are needed to discriminate if a signal is linked to progression or to the acquisition phenotype.

#### The ACS group

417 HIV-1 seroconverter and seroprevalent Dutch subjects were collected from the ACS (Amsterdam Cohort Study) on the course of HIV-1 infection using AIDS-related death as an endpoint [50]. AIDS-related death is defined as death with AIDS-related malignancy, death with AIDS-opportunistic infections, or death with an AIDS-related cause not specified by the treating physician. Written informed consent was obtained prior to data collection for the ACS, and the study was approved by the Academic Medical Center institutional medical ethics committee.

#### The MACS cohort

156 HIV-1 seroconverter Caucasian homosexual men were collected from the MACS (Multicenter AIDS Cohort Study) cohort using time to clinical AIDS (CDC 1987 definition) as an endpoint [51]. This panel was enriched with extreme AIDS progression phenotypes (rapid progressors, and long-term non-progressors) in order to increase the power of the study.

### Genotyping methods and quality control for the *CD39* genetic association study

(For more details, see previously published works [23], [50], [51]). For the GRIV (cases and controls) and ACS analyses, the *CD39* genotyping data were obtained using the Illumina Infinium II HumanHap300 BeadChips, when for the MACS analysis, they were obtained using the Affymetrix GeneChip Human Mapping 500K Array. In each study, quality control filters (*e.g.* missingness, low minor allele frequency, Hardy-Weinberg equilibrium deviation) were applied to ensure reliable genotyping data as previously described [23], [50], [51]. In each cohort, potential population stratification was also considered using the Eigenstrat software [52]. First, to confirm continental ancestries, the genotypes of each participants group were combined with the genotypes from the three HapMap reference populations. Among the initial ACS group, 13 subjects were thus excluded from further analyses (n = 404) to avoid spurious associations resulting from a non-European ancestry. Then, in each study group of European descent, the top ten most significant principal components were identified and included as covariates in the regression models described below. The rs11188513 SNP untyped in the MACS group was imputed using Impute software [53] and the HapMap release 21 phased data for the population of European descent (CEU) as the reference panel.

### Statistical analysis for the *CD39* genetic association study

We first performed a genetic case-control association analysis in the GRIV cohort using a logistic regression and an additive model, including as covariates the 10 principal components identified by Eigenstrat. All SNPs found to be significant in the GRIV cohort were tested for replication in ACS and MACS cohorts. The SNP rs11188513 was the only polymorphism exhibiting a significant p-value both in ACS and MACS. For the replication in the ACS and MACS groups, we performed Kaplan-Meier survival analysis and regression -Cox proportional regression and linear regression for ACS and MACS respectively- in an additive model including as covariates the 10 principal components identified by Eigenstrat. The significant associations (*P*<0.05) were also retested using age, sex, and *CCR5-P1* and *D32* polymorphisms as covariates and yielded identical results.

To evaluate the combined *p-value* obtained over the 3 cohorts for each SNP, we used the classical Fisher method [54].

### Ethic statements

Approval and written informed consent from all subjects were obtained before study initiation. The study was approved by the following ethical committees : Hospital of Infectious Diseases, Sofia, Bulgaria and CCP IX Ile de France - Henri Mondor Hospital, Créteil, France. Ethic statements for GRIV, MACS ACS cohorts have been already reported [23], [50], [51].

## Supporting Information

Figure S1 Expression of CD39 on CD4+CD25^high^FoxP3+CD127^low^ Treg and CD4+CD25^low^ cells. **(a)** gating strategy: Representative experiment showing the expression of CD39 on Treg and CD4+CD25^low^ activated T cells from an HIV-negative donor. **(b** and **c)** PBMC from c-ART− HIV-1 positive patients (black squares, n = 39), c-ART+ HIV-positive subjects (grey squares, n = 39) and HIV- negative controls (white squares, n = 25) were analysed by flow cytometry. Percentages of CD4+CD25^low^ **(b)** CD4+CD25^low^CD39+ **(c)** are shown. Statistical differences were assessed by unpaired t-test assuming independent samples * P<0.05, ** P<0.01, ***P<0.001).
(PPT)

Figure S2 Phenotype of Treg CD39+. CD3 T cells purified from PBMC from c-ART− (black histogram, n = 8) and c-ART+ (grey histogram, n = 7) HIV-positive subjects, and from HIV-negative controls (white histogram n = 7). The distribution of naïve (CD45RA+CD28−) central memory (CD45RA−CD28+) effector memory (CD45RA−CD28−) and terminal effector cells (CD45RA+CD28−) among CD4+CD25highCD127lowFoxP3+ Treg CD39+, are represented in **(a).** The expression of CD39 on Treg CD28+CD45RA+ and Treg CD28+CD45RA− subsets is represented in **(b).** Statistical differences were assessed by unpaired t-test assuming independent samples, * P<0.05.
(PPT)

Figure S3 Mechanism of action of CD39 mAb BY40. **(a)** Down-regulation of CD39 molecule induced by CD39 mAb BY40. Histogram overlays represent CD39 expression on the surface of YT2C2 NK line cells before (thick line) and after 2 h of culture in the presence of BY40 (dotted line), in comparison to cells stained with an irrelevant isotype control (filled histogram). This result is representative of three independent experiments. **(b)** CD39 mAb BY40 inhibits the ATPase activity of CD39. Monocytes were cultured alone or in the presence of BY40 or control IgG1 mAb (10 µg/mL) for 16 h. The cells were then washed with a phosphate-free reaction buffer (containing 0.5 mM CaCl2, 120 mM NaCl, 5 mM KCl, 60 mM glucose, and 50 mM Tris −HCl buffer, pH = 8) and ATPase activity was initiated by the addition of ATP at a concentration 100 M in 200 µl of reaction buffer for 15 min at 37°C. The release of inorganic phosphate was measured using the malachite green phosphate detection kit (R&D System, Minneapolis, USA) according to the manufacturer's instructions. This result is representative of three independent experiments. * P<0.05.
(PPT)

Figure S4 Competitivity test experiment between commercial anti-CD39 (clone TÜ66) and BY40. Purifed CD4 T cells stained at 4°c during 30 min. with PE anti-CD39 (clone TÜ66) with or without BY40 at 10 µg/ml. CD39 expression was gated on CD4+CD25high.
(PPT)

Figure S5 Genetic map of *CD39* gene. Exons and introns are symbolized by full and empty rectangles, respectively. The positions of the ATG and STOP cordons are indicated by a triangle (▸) and by an asterisk (*), respectively. The significant polymorphisms in the GRIV study are indicated by the symbol. Of note, an alternative splicing variant has been described that differs from the one presented in the position of the first exon.
(PPT)

Figure S6 Expression of CD39 on NK cells, B cells, monocytes and CD8+ T cells. **(a)** Representative experiment showing the expression of CD39 on NK cells, B cells, monocytes and CD8+ T cells from an HIV-negative and a c-ART−HIV-positive patient. **(b)** Cumulative data of CD39 expression on monocytes and B cells (MFI) and CD8+ T cells and NK cells (%) from c-ART−HIV-positive (n = 3), c-ART+HIV-positive (n = 3) and HIV negative (n = 3) donors.
(PPT)

Table S1 Allelic frequencies in the different populations. the French GRIV cohort (LTNP: long-term non-progressor ; CTR: seronegative controls ; RP: rapid progressors), the Dutch ACS, and the USA MACS. Unlike the GRIV cohort, ACS and MACS are seroconverter cohorts containing subjects with all type of progression profiles. The frequency in these two cohorts are thus naturally similar to these observed in the control groups, and the progression effect is observed through Kaplan-Meier curves (see Fig. 7).
(DOCX)
