# Supplementary material for: CD39/Adenosine Pathway Is Involved in AIDS Progression
Source: PLoS Pathog. 2011 Jul 7;7(7):e1002110. doi: 10.1371/journal.ppat.1002110 (PMC3131268; doi:10.1371/journal.ppat.1002110)
Supplement: Figure S1 — Expression of CD39 on CD4+CD25highFoxP3+CD127low Treg and CD4+CD25low cells. (a) gating strategy: Representative experiment showing the expression of CD39 on Treg and CD4+CD25low activated T cells from an HIV-negative donor. (b and c) PBMC from c-ART− HIV-1 positive patients (black squares, n = 39), c-ART+ HIV-positive subjects (grey squares, n = 39) and HIV- negative controls (white squares, n = 25) were analysed by flow cytometry. Percentages of CD4+CD25low (b) CD4+CD25lowCD39+ (c) are shown. Statistical differences were assessed by unpaired t-test assuming independent samples * P<0.05, ** P<0.01, ***P<0.001). (PPT) [file ppat.1002110.s001.ppt]

## Slide 1
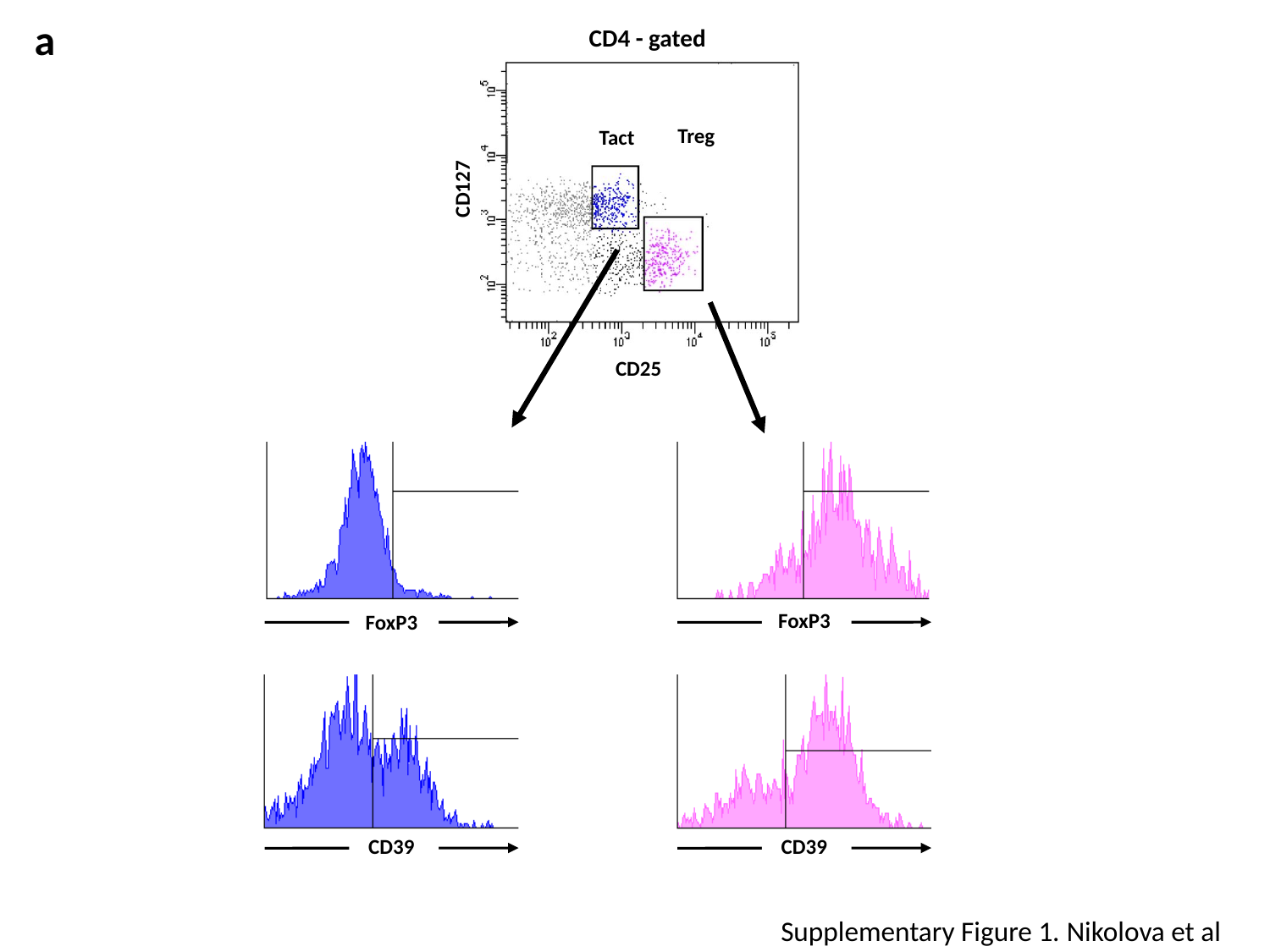

a
CD4 - gated
Treg
Tact
CD127
CD25
FoxP3
FoxP3
CD39
CD39
Supplementary Figure 1. Nikolova et al

## Slide 2
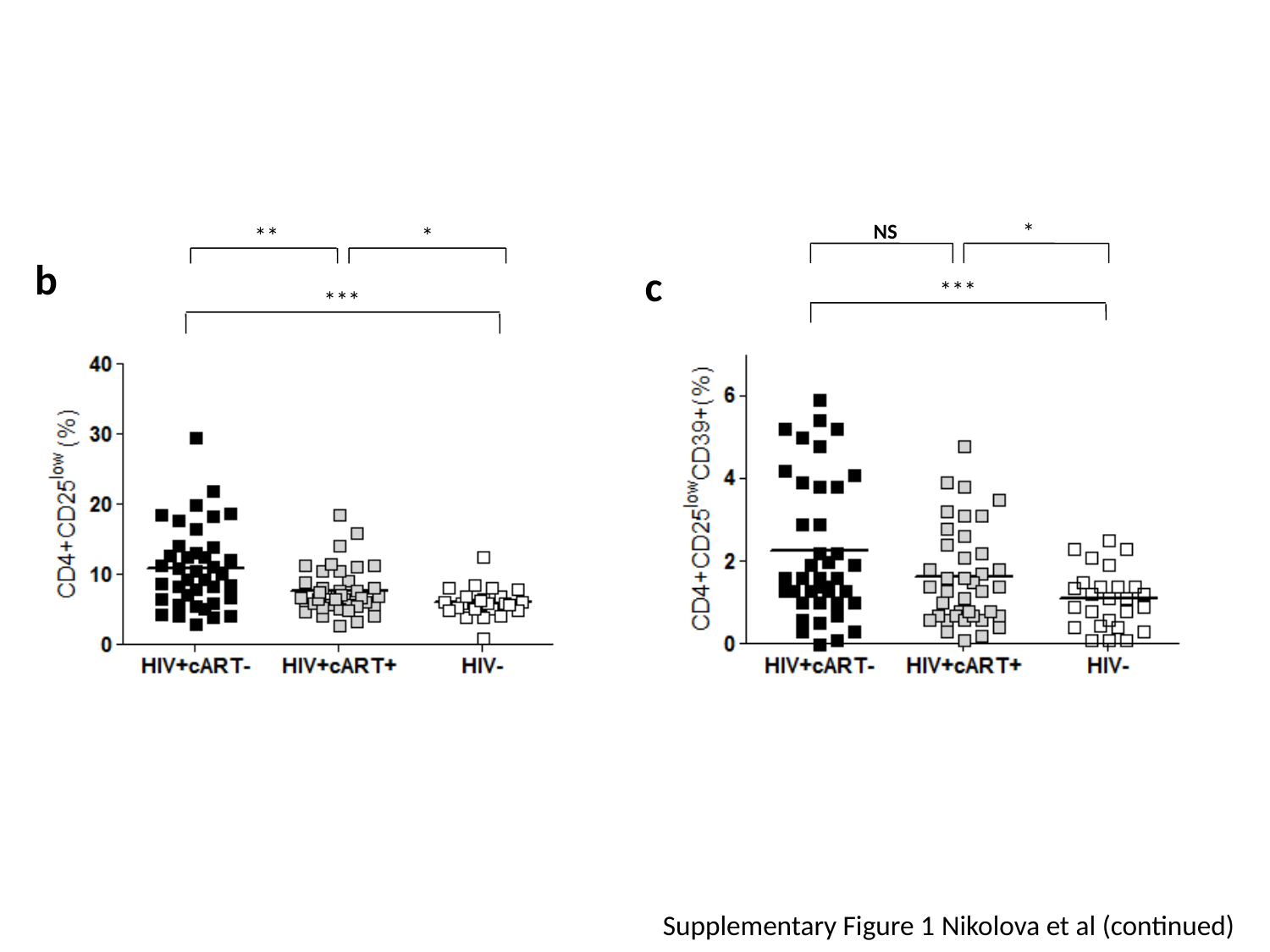

*
NS
***
**
 *
***
b
c
Supplementary Figure 1 Nikolova et al (continued)
