# Supplementary material for: CD39/Adenosine Pathway Is Involved in AIDS Progression
Source: PLoS Pathog. 2011 Jul 7;7(7):e1002110. doi: 10.1371/journal.ppat.1002110 (PMC3131268; doi:10.1371/journal.ppat.1002110)
Supplement: Figure S2 — Phenotype of Treg CD39+. CD3 T cells purified from PBMC from c-ART− (black histogram, n = 8) and c-ART+ (grey histogram, n = 7) HIV-positive subjects, and from HIV-negative controls (white histogram n = 7). The distribution of naïve (CD45RA+CD28−) central memory (CD45RA−CD28+) effector memory (CD45RA−CD28−) and terminal effector cells (CD45RA+CD28−) among CD4+CD25highCD127lowFoxP3+ Treg CD39+, are represented in (a). The expression of CD39 on Treg CD28+CD45RA+ and Treg CD28+CD45RA− subsets is represented in (b). Statistical differences were assessed by unpaired t-test assuming independent samples, * P<0.05. (PPT) [file ppat.1002110.s002.ppt]

## Slide 1
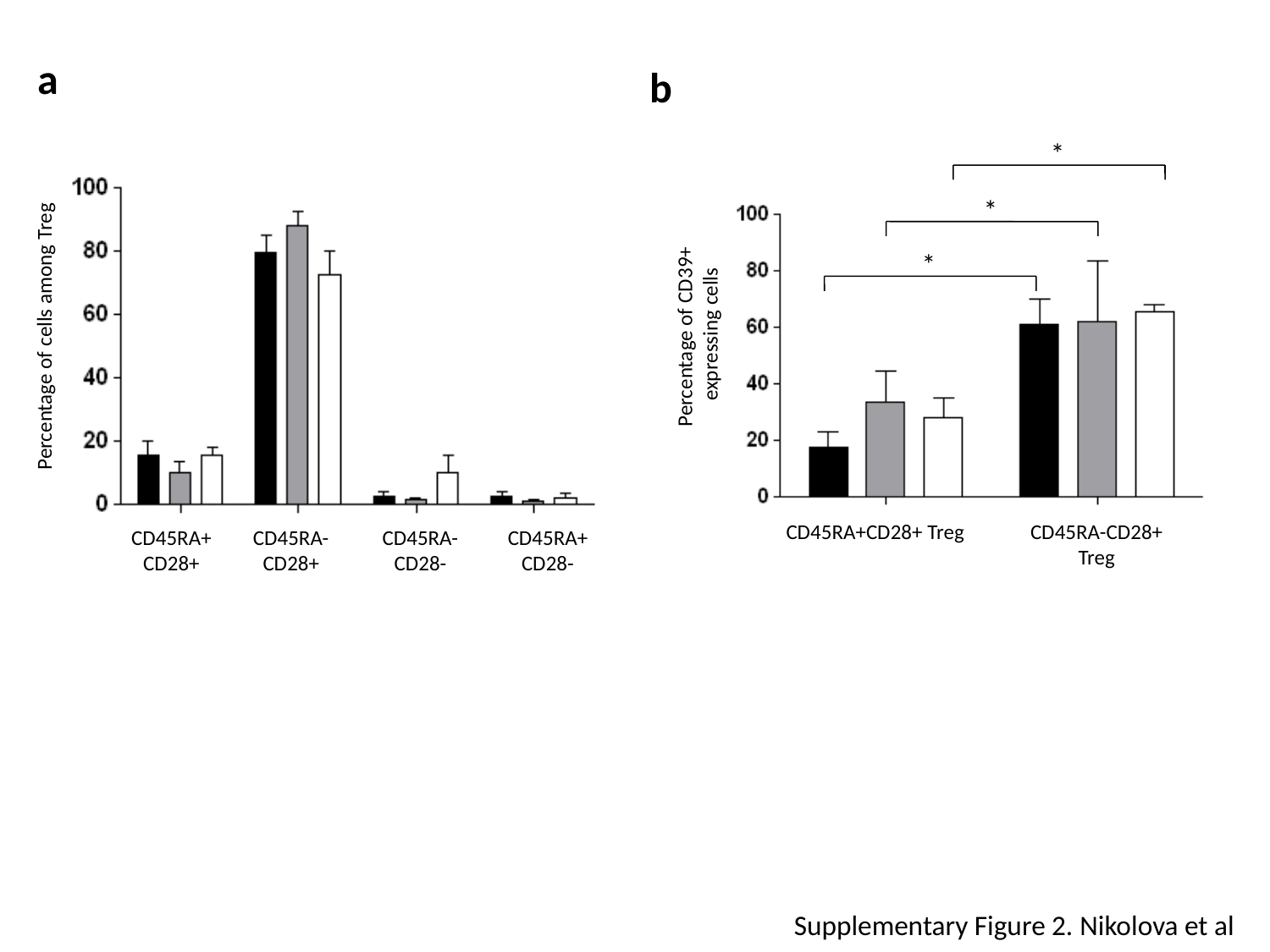

a
b
*
*
*
Percentage of CD39+
expressing cells
Percentage of cells among Treg
CD45RA+CD28+ Treg
CD45RA-CD28+
Treg
CD45RA+
CD28+
CD45RA-
CD28+
CD45RA-
CD28-
CD45RA+
CD28-
Supplementary Figure 2. Nikolova et al
