# Supplementary material for: CD39/Adenosine Pathway Is Involved in AIDS Progression
Source: PLoS Pathog. 2011 Jul 7;7(7):e1002110. doi: 10.1371/journal.ppat.1002110 (PMC3131268; doi:10.1371/journal.ppat.1002110)
Supplement: Figure S3 — Mechanism of action of CD39 mAb BY40. (a) Down-regulation of CD39 molecule induced by CD39 mAb BY40. Histogram overlays represent CD39 expression on the surface of YT2C2 NK line cells before (thick line) and after 2 h of culture in the presence of BY40 (dotted line), in comparison to cells stained with an irrelevant isotype control (filled histogram). This result is representative of three independent experiments. (b) CD39 mAb BY40 inhibits the ATPase activity of CD39. Monocytes were cultured alone or in the presence of BY40 or control IgG1 mAb (10 µg/mL) for 16 h. The cells were then washed with a phosphate-free reaction buffer (containing 0.5 mM CaCl2, 120 mM NaCl, 5 mM KCl, 60 mM glucose, and 50 mM Tris −HCl buffer, pH = 8) and ATPase activity was initiated by the addition of ATP at a concentration 100 M in 200 µl of reaction buffer for 15 min at 37°C. The release of inorganic phosphate was measured using the malachite green phosphate detection kit (R&D System, Minneapolis, USA) according to the manufacturer's instructions. This result is representative of three independent experiments. * P<0.05. (PPT) [file ppat.1002110.s003.ppt]

## Slide 1
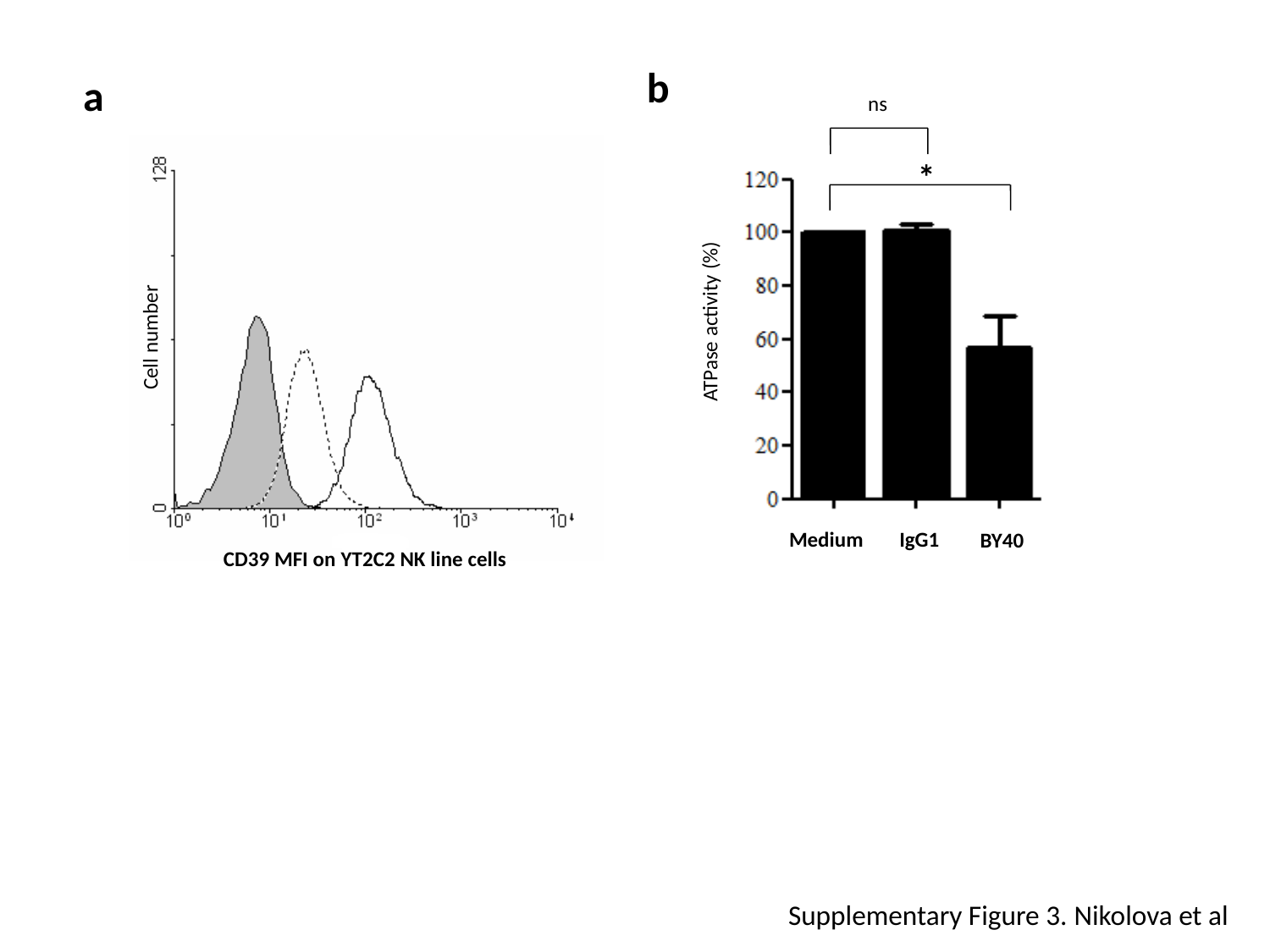

ns
*
ATPase activity (%)
Medium
IgG1
BY40
b
a
Cell number
CD39 MFI on YT2C2 NK line cells
Supplementary Figure 3. Nikolova et al
