# Supplementary figures and images for: CD39/Adenosine Pathway Is Involved in AIDS Progression
Source: PLoS Pathog. 2011 Jul 7;7(7):e1002110. doi: 10.1371/journal.ppat.1002110 (PMC3131268; doi:10.1371/journal.ppat.1002110)

## Slide 1
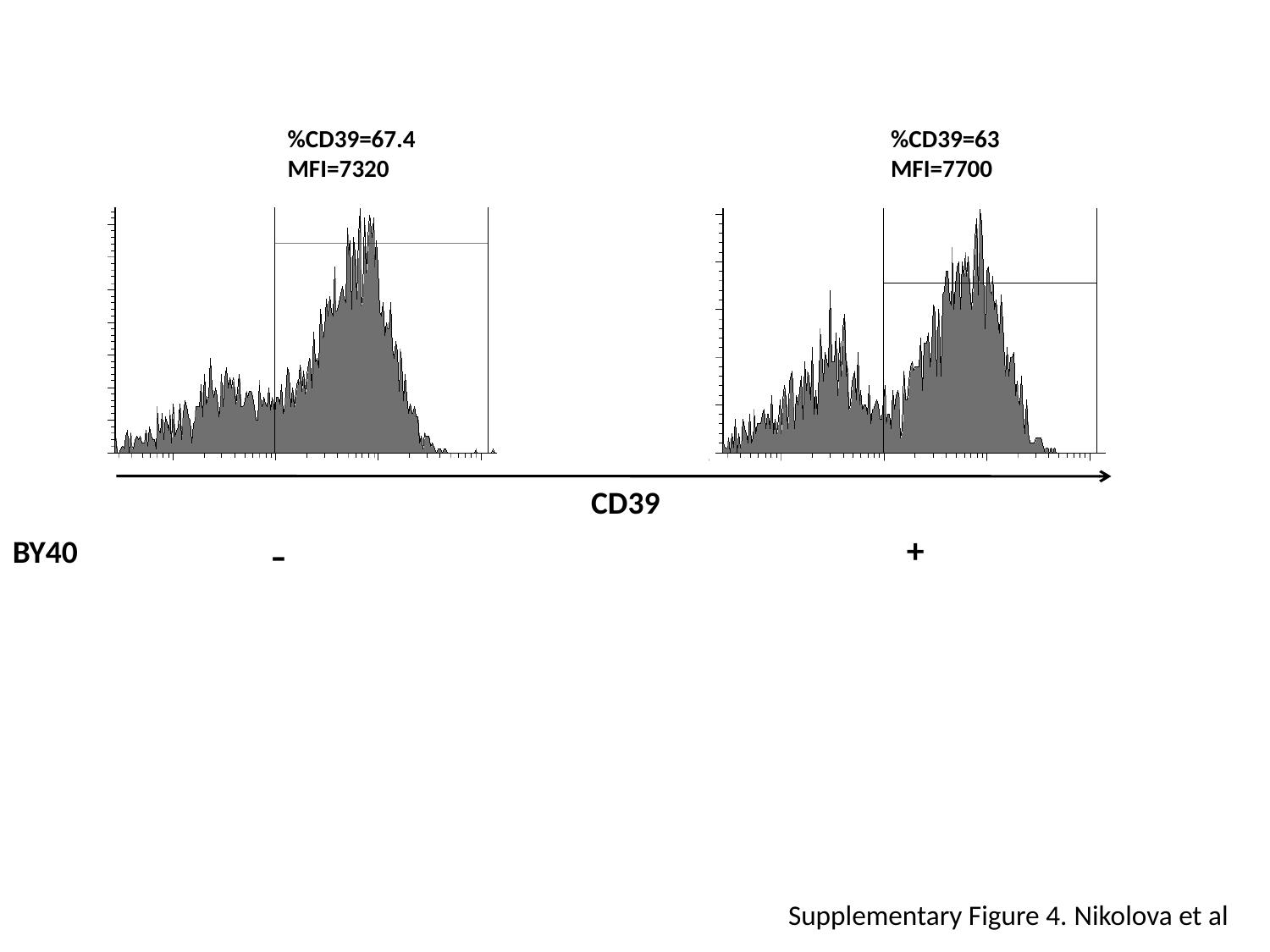

%CD39=67.4
MFI=7320
%CD39=63
MFI=7700
CD39
-
+
BY40
Supplementary Figure 4. Nikolova et al

Supplement: Figure S4 — Competitivity test experiment between commercial anti-CD39 (clone TÜ66) and BY40. Purifed CD4 T cells stained at 4°c during 30 min. with PE anti-CD39 (clone TÜ66) with or without BY40 at 10 µg/ml. CD39 expression was gated on CD4+CD25high. (PPT) [file ppat.1002110.s004.ppt]

## Slide 1
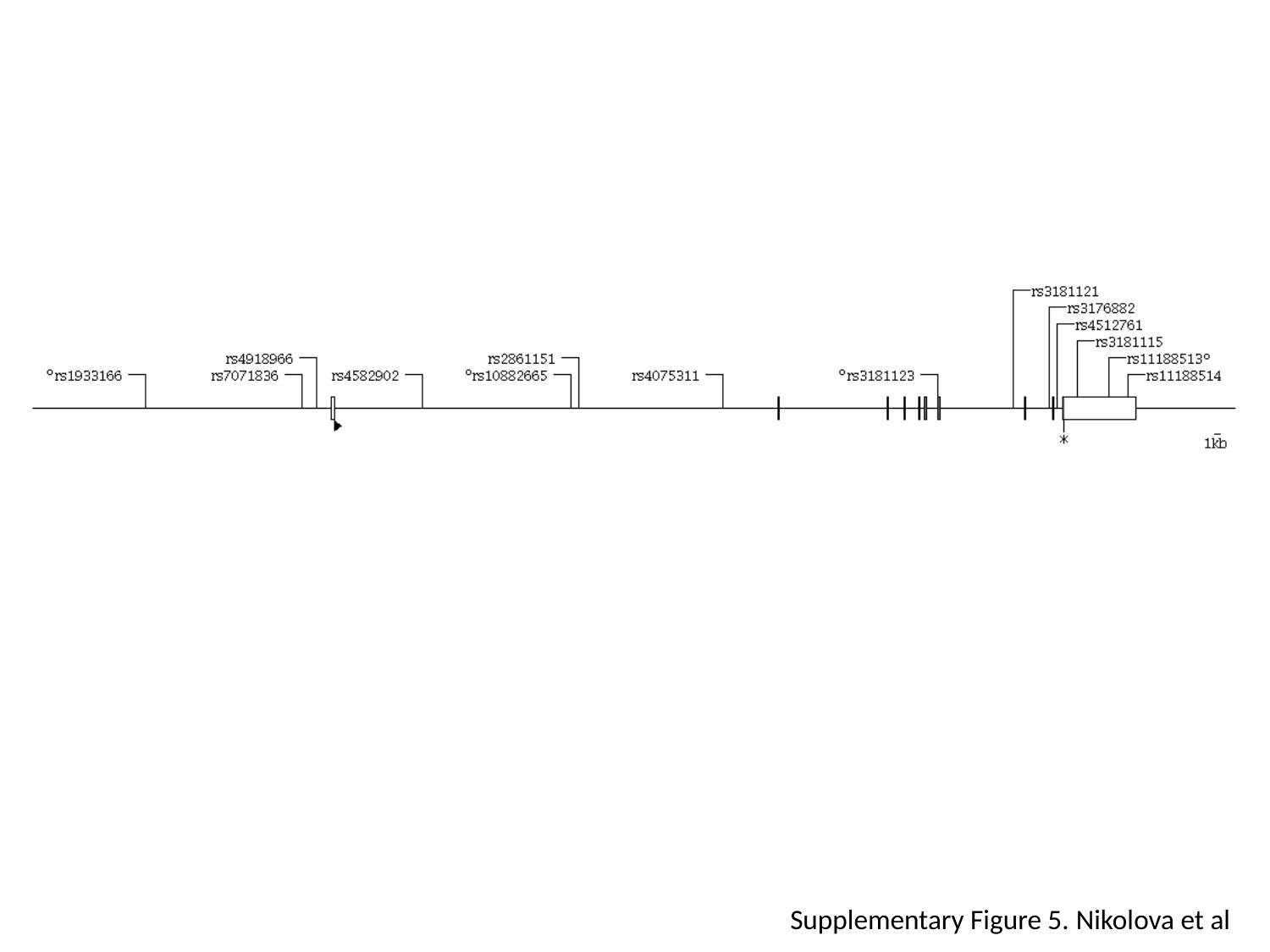

Supplementary Figure 5. Nikolova et al

Supplement: Figure S5 — Genetic map of CD39 gene. Exons and introns are symbolized by full and empty rectangles, respectively. The positions of the ATG and STOP cordons are indicated by a triangle (▸) and by an asterisk (*), respectively. The significant polymorphisms in the GRIV study are indicated by the symbol. Of note, an alternative splicing variant has been described that differs from the one presented in the position of the first exon. (PPT) [file ppat.1002110.s005.ppt]

## Slide 1
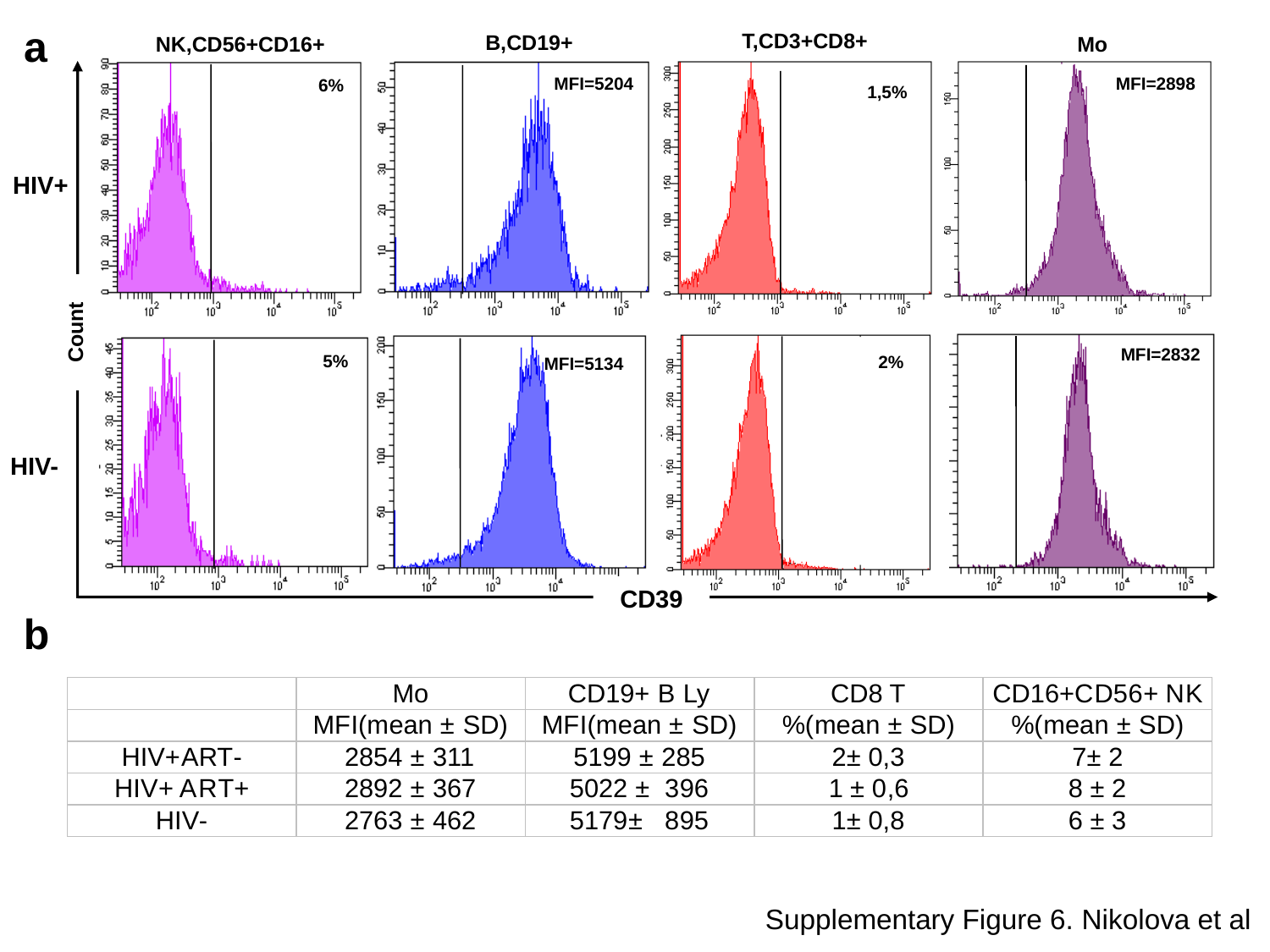

a
T,CD3+CD8+
B,CD19+
NK,CD56+CD16+
Mo
MFI=5204
MFI=2898
6%
1,5%
HIV+
Count
MFI=2832
5%
2%
MFI=5134
HIV-
CD39
b
Supplementary Figure 6. Nikolova et al

Supplement: Figure S6 — Expression of CD39 on NK cells, B cells, monocytes and CD8+ T cells. (a) Representative experiment showing the expression of CD39 on NK cells, B cells, monocytes and CD8+ T cells from an HIV-negative and a c-ART−HIV-positive patient. (b) Cumulative data of CD39 expression on monocytes and B cells (MFI) and CD8+ T cells and NK cells (%) from c-ART−HIV-positive (n = 3), c-ART+HIV-positive (n = 3) and HIV negative (n = 3) donors. (PPT) [file ppat.1002110.s006.ppt]
