# Supplementary material for: CD39/Adenosine Pathway Is Involved in AIDS Progression
Source: PLoS Pathog. 2011 Jul 7;7(7):e1002110. doi: 10.1371/journal.ppat.1002110 (PMC3131268; doi:10.1371/journal.ppat.1002110)
Supplement: Table S1 — Allelic frequencies in the different populations. the French GRIV cohort (LTNP: long-term non-progressor ; CTR: seronegative controls ; RP: rapid progressors), the Dutch ACS, and the USA MACS. Unlike the GRIV cohort, ACS and MACS are seroconverter cohorts containing subjects with all type of progression profiles. The frequency in these two cohorts are thus naturally similar to these observed in the control groups, and the progression effect is observed through Kaplan-Meier curves (see Fig. 7). (DOCX) [file ppat.1002110.s007.docx]

|  | **GRIV LTNP** | **GRIV CTR** | **GRIV RP** | **ACS** | **Dutch CTR** | **MACS** | **USA CTR** |
| --- | --- | --- | --- | --- | --- | --- | --- |
| **rs11188513-C** | **39%** | **34%** | **35%** | **32%** | **32%** | **35%** | **36%** |
